# Metaverse-Aided Rehabilitation: A Perspective Review of Successes and Pitfalls

**DOI:** 10.3390/jcm14020491

**Published:** 2025-01-14

**Authors:** Michele Vecchio, Rita Chiaramonte, Enrico Buccheri, Sofia Tomasello, Pierfrancesco Leonforte, Antonio Rescifina, Antonio Ammendolia, Umile Giuseppe Longo, Alessandro de Sire

**Affiliations:** 1 Department of Biomedical and Biotechnological Sciences, University of Catania, 95123 Catania, Italy; michele.vecchio@unict.it (M.V.); rita.chiaramonte@unict.it (R.C.); enrico.buccheri@gmail.com (E.B.); pierfra.leonforte@hotmail.it (P.L.); 2 Rehabilitation Unit, AOU Policlinico G. Rodolico-San Marco, 95123 Catania, Italy; 3 Faculty of Medicine and Surgery, University of Palermo, 90127 Palermo, Italy; sofia.tomasello@studenti.univr.it; 4 Department of Drug and Health Sciences, University of Catania, Viale Andrea Doria, 95125 Catania, Italy; larescifina@unict.it; 5 Physical Medicine and Rehabilitation Unit, Department of Medical and Surgical Sciences, University of Catanzaro “Magna Graecia”, 88100 Catanzaro, Italy; ammendolia@unicz.it (A.A.); alessandro.desire@unicz.it (A.d.S.); 6 Research Center on Musculoskeletal Health, MusculoSkeletalHealth@UMG, University of Catanzaro “Magna Graecia”, 88100 Catanzaro, Italy; 7 Fondazione Policlinico Universitario Campus Bio-Medico, Via Alvaro del Portillo 200, 00128 Roma, Italy; 8 Research Unit of Orthopaedic and Trauma Surgery, Department of Medicine and Surgery, Università Campus Bio-Medico di Roma, Via Alvaro del Portillo 21, 00128 Roma, Italy

**Keywords:** metaverse, rehabilitation, artificial intelligence, virtual reality, technology

## Abstract

**Background**: The evolution of technology has continuously redefined the landscape of rehabilitation medicine. Researchers have long incorporated virtual reality (VR) as a promising intervention, providing immersive therapeutic environments for patients. The emergence of the metaverse has recently further expanded the potential applications of VR to augment the possibilities in rehabilitation. Rehabilitation is a crucial aspect of healthcare, and technological advancements have allowed new approaches to aid in this process. One such approach is the metaverse, a virtual world where users can interact with each other and their surroundings in a simulated environment. This comprehensive review aimed to analyze the scientific evidence using the term “metaverse” in rehabilitation and its potential patient benefits. **Methods**: We conducted a comprehensive literature search from the inception to September 2024 in PubMed, Scopus, Web of Science, and Cochrane Database to identify studies investigating the term “metaverse” and its role in rehabilitation. We then assessed these studies based on their methodology, patient population, technology used, and therapeutic outcomes. **Results**: Out of 81 articles, 55 remained after removing duplicates. After screening the title, abstract, and full text, we included five articles. **Conclusions**: Results from these studies suggested potential benefits in various rehabilitative areas, such as cerebral palsy, intellectual disabilities, pain management, and physical performance improvement among the elderly. The metaverse presents promising avenues for enhancing rehabilitation outcomes. While VR’s effectiveness is well established, the metaverse, being a newer concept, necessitates further studies for a more comprehensive understanding.

## 1. Introduction

Virtual reality (VR) has ventured into medicine and education as technology progresses. The metaverse builds upon VR’s immersion, fusing virtual environments, augmented reality (AR), and online platforms. This fusion transformed digital experiences into a cohesive and shared virtual universe, called metaverse. Accessed through VR headsets, it employs avatars and blockchain to bridge physical and virtual realms seamlessly [1], emerging as a future mobile computing platform [2].

Recently, Yang et al. proposed the metaverse concept in medicine, defined as the Medical Internet of Things (MIoT) using AR and VR technologies [3,4]. VR and the metaverse revolve around digital spaces and virtual experiences but differ in scope, purpose, and complexity. In particular, VR is well established in rehabilitation, whereas the metaverse is gaining traction in various domains for its broader interactive potential. The metaverse comprises four key elements: AR, lifelogging, mirror world, and VR. AR overlays digital graphics onto our real environment, exemplified by tools like Pokemon 3D and Google Glasses [5]. Lifelogging employs smart devices to chronicle and share daily activities online. The mirror world virtually replicates real-world data, seen in platforms like Google Maps. Conversely, VR creates an immersive 3D environment where users can customize avatars and interact in virtual spaces, akin to online multiplayer games and virtual hospitals [5].

Wang et al. [6] advocate for a “medical technology and AI” (MeTAI) metaverse, integrating virtual comparative scans, personalized patient avatars, and secure access to medical data, with the aim to enhance current VR, AR, and telemedicine standards.

The most important impact of the metaverse will be on how physicians and patients utilize medical data and apply tools for disease diagnosis, therapy selection, and implementation, such as in cases of surgery and other treatments. The metaverse provides immersive simulations for medical students and professionals, allowing hands-on practice in a risk-free virtual environment. Examples include surgical training using VR and collaborative learning spaces. Indeed, in the metaverse, surgeons or doctors can test different therapies on avatars, optimizing treatment plans through patient-specific computer simulations and refining skills in risk-free settings. This practice makes the metaverse applicable to a wide range of medical interventions, offering a personalized and precise approach to healthcare [6]. Furthermore, the metaverse supports cognitive and physical rehabilitation through immersive simulations tailored to patient-specific needs, also with remote access therapy in virtual environments; thus, the metaverse can be adopted for collaborative learning, multi-institutional projects, and team training through metaverse interactions similar to those in the real world [1].

Recent systematic reviews with meta-analyses showed that VR might play a role in functioning in patients affected by several neurological diseases of rehabilitative interest (e.g., stroke, multiple sclerosis, and Parkinson’s disease [7,8,9,10,11,12] and in cognitive disorders and psychiatric fields [13,14,15]. Moreover, in cancer care, AI has been shown to aid in rehabilitating these subjects [16]. However, limited studies have explored the metaverse’s role in medicine and rehabilitation. While it has been used for social integration at school [17] or the removal of architectural barriers [18], its introduction to the rehabilitation field is a recent development, starting only in 2022 [19].

Despite its potential, several gaps remain about (1) its long-term efficacy and its effectiveness in achieving sustainable outcomes, especially in rehabilitation, a topic not well documented yet; (2) its accessibility to diverse populations, including those in low-resource settings; (3) ethical concerns like data privacy, informed consent in virtual environments, and psychological impacts of prolonged metaverse use that require further exploration; (4) the cost-effectiveness of technologies in healthcare; (5) the possibility of the integration of metaverse platforms with existing healthcare systems and electronic health records; and (6) the impact on healthcare providers, because little is known about how these technologies affect the workload, mental health, and skill requirements of healthcare professionals and patients.

Currently, there is a scarcity of high-impact studies, such as RCTs, exploring rehabilitation through the metaverse. Future research should aim to contrast the efficacy of metaverse-based rehabilitation with conventional VR/AR interventions and placebo-controlled VR/AR.

Overall, while the metaverse’s use in rehabilitation shows significant promise, it is essential to carefully consider its potential benefits and drawbacks before implementing it as standard practice. Conducting more rigorous studies will be crucial to fully understand its efficacy and address the challenges of integrating virtual and real-world rehabilitation outcomes.

Therefore, this narrative review aimed to investigate the role of the metaverse in rehabilitation to better understand its potential applications in clinical practice.

## 2. Materials and Methods

### Search Strategy

This narrative review followed the SANRA quality criteria [20]. A comprehensive literature research was conducted from the inception to September 2024 on PubMed, Scopus, Cochrane Database, and Web of Science, using the search string “metaverse” AND “rehabilitation” and the related term “rehabilitative”, to analyze the scientific evidence using the term “metaverse” in the rehabilitation field and its potential benefits for patients. The inclusion criteria included (a) any type of human trial, ranging from randomized controlled trials (RCTs) to prospective and retrospective studies, that focuses on the application of the metaverse in rehabilitation; (b) published in the English language; (c) no restrictions on publication date.

We excluded reviews, letters to the editor, expert opinions, interviews, and studies which were not relevant to the central theme of this review.

Then, data extraction and synthesis were performed using a qualitative method. Two authors independently extracted and synthesized information on the role of the metaverse in rehabilitation. In cases of disagreement, the opinion of a third author was asked. The heterogeneity of the collected data, the different ages and pathologies of the included patients, and the limited number of studies precluded conducting a quantitative and qualitative data analysis.

## 3. Results

Out of 81 articles generated by the electronic search, 55 records remained after removing duplicates. After the title, abstract, and full-text screening, five articles were included and assessed in the present narrative review.

### 3.1. Characteristics of the Included Studies

Five studies were included in the review: 1. An RCT comparing the metaverse, traditional treatment, and control (no intervention) for rehabilitation in intellectual disabilities [21]. 2. An RCT evaluating the metaverse versus physiotherapy for cerebral palsy [22]. 3. A retrospective study on the metaverse’s application for non-specific low back pain (NS-LBP) and/or neck pain disorders (NPDs) [23]. 4. A prospective study exploring the use of the metaverse for exercise therapy in elderly people [24]. 5. An RCT exploring the use of the metaverse for exercise therapy in young people [25].

The total number of participants across the studies was 315, and further details are provided below and in Table 1.

### 3.2. Metaverse in Intellectual Disability Rehabilitation

The RCT by Cheung et al. [21] employed VR wearable technology, akin to the metaverse, for human–virtual world interaction. In total, 145 intellectually disabled participants were grouped into VR (n = 42), traditional (n = 53), and control (no intervention) (n = 50). The study used various scales to assess shopping, cooking, and kitchen cleaning skills. After accounting for age, gender, and intelligence quotient, the VR group significantly outperformed in cooking and cleaning tasks. It exhibited enhanced memory compared to other groups, with IQ being a notable confounding factor.

### 3.3. Metaverse and Cerebral Palsy

The RCT by Moon et al. [22] evaluated the efficacy of metaverse physical therapy (MPT) versus conventional physical therapy (CPT) in children with cerebral palsy. In total, 26 participants were randomized into MPT or CPT, undergoing therapy thrice weekly for four weeks. Measurements included the Gross-Motor-Function Measure 66 (GMFM-66), heart rate, perceived exertion, functional independence, quality of life, and COVID-19 transmission risk. MPT demonstrated superior results in GMFM-66, heart rate, and exertion scores and showed a notably reduced perceived risk of COVID-19 transmission compared to CPT.

### 3.4. Exercise Therapy for NS-LBP and NPDs in the Metaverse

Orr et al.’s retrospective study [23] assessed the efficacy of metaverse-delivered therapeutic exercise in treating 82 subjects with non-specific low back pain (NS-LBP) and/or neck pain disorders (NPDs). They observed a significant 17.8% reduction in NS-LBP disability and 23.2% in NPDs. The metaverse-based therapeutic exercise was feasible and safe, with no reported adverse events.

### 3.5. Exercise Therapy for Young and Elderly People in the Metaverse

The RCT by Mizuta et al. [25] demonstrated that the metaverse space facilitated the delivery of exercise videos to encourage increased physical activity. The analysis revealed a notable increase in total physical activity among the metaverse group, consisting of 16 young individuals, after the intervention compared to before. In contrast, no significant changes were observed in the 16 participants of the YouTube group and another 16 in the control group, who did not receive specific instructions to engage in sports.

Shah et al.’s prospective study [24] used a Social VR-based collaborative exergame to encourage exercise among 14 elderly participants over five weeks. Participants were split into collaborative and individual play groups. By the fourth week, those in collaborative sessions displayed significantly increased motivation and physical exertion compared to solo players. Collaborative VR gameplay proved highly motivating, with reported health benefits, minimal simulator sickness, and high usability scores.

## 4. Discussion

**Figure 1:**
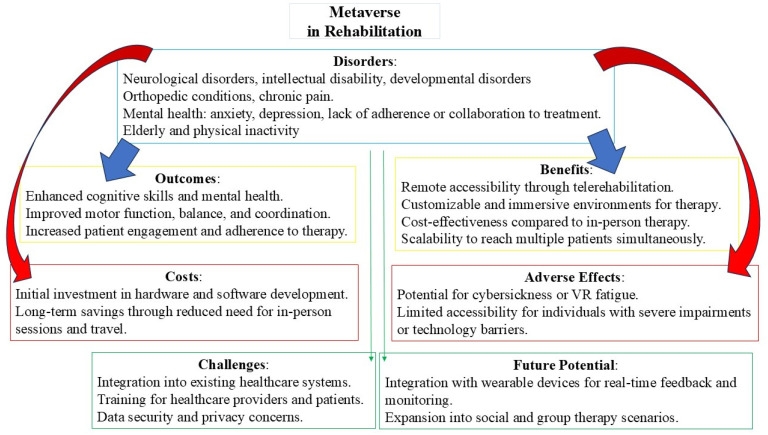
Key points of metaverse in rehabilitation.

This review evaluates the metaverse’s potential in rehabilitation, drawing from four studies that address conditions like intellectual disability [21], cerebral palsy, neck and lumbar pain [23], the physical decline in the elderly [24], and maintaining good physical performance in younger [25] (see Figure 1 for further details).

More specifically, VR, with its immersive environments, has influenced the rehabilitation field, enabling tailored physical and cognitive exercises for various conditions, such as stroke and all the conditions with impairment of mobility [26] and language involvement [27]. The software used for language evaluation can develop even further in processing and data integration thanks to artificial intelligence in rehabilitation settings, potentially revolutionizing the speech therapy interventions by providing more precise, personalized, and practical solutions. Building upon VR’s principles, the metaverse aims to reshape digital interactions and experiences in rehabilitation (Table 2). However, despite VR being more established in rehabilitation, the metaverse is gaining traction in various rehabilitation domains for its broader interactive potential (Table 3).

These promising findings align with earlier VR or AR research [28,29,30,31], suggesting that the metaverse could further enhance therapeutic effects. However, the limited sample size (n. 267) and varied study designs restrict a definitive comparison of the metaverse’s efficacy to traditional methods. Using the metaverse in rehabilitation integrates VR and AR with therapeutic methods. These immersive settings hold promise for physical, cognitive, and psychological rehabilitation by offering engaging, controlled environments for patients.

### 4.1. VR and AR in the Rehabilitation Field

For decades, VR and AR have enhanced rehabilitation outcomes, with numerous studies highlighting their efficacy in neurological, orthopedic, geriatric, and pediatric conditions [29], especially in stroke rehabilitation [29,31,32,33], chronic neck pain, shoulder impingement syndrome, rheumatoid arthritis, knee arthritis, ankle instability, post-anterior cruciate reconstruction [30], imbalance in the elderly, and Parkinson’s disease [33].

### 4.2. Metaverse in Rehabilitation: Efficacy and Costs

The emergence of the metaverse, a fusion of VR and AR, prompts exploration into its efficacy and cost-effectiveness, especially when integrated with advanced diagnostic tools [6]. The scalability and cost-effectiveness of the metaverse in rehabilitation depend on several factors, including technological accessibility, integration into healthcare systems, and evidence-based outcomes. Its immersive nature uniquely suits rehabilitation, enriching patients’ sensory experiences [28]. Multisensory technologies match standard treatments in efficacy, especially for cognitive aspects [28]. Noteworthy research emphasizes the transformative role of VR and AR in medicine, particularly rehabilitation [7,16,34]. Concurrently, other studies underscore the metaverse’s promising avenues, inherent potential, and rehabilitation focus, especially concerning neurodegenerative disorders [35], interventions for cognitive decline [36], and multisensory cognitive rehabilitation through the metaverse’s sensory-rich environment [28].

**Table 3:** Application of virtual reality in rehabilitation.

Rehabilitation Field	Virtual Reality	Metaverse
**Physical Rehabilitation**	VR can simulate physical activities, allowing patients to practice movements and exercises in a controlled environment. VR has beneficial effects in several health conditions, particularly in alleviating pain (41%), enhancing functional ability (31%), and improving muscle strength (24%) [11].	The metaverse through physical therapy can improve the quality of life of patients with NS-LBP (improving by 17.8% at the Modified Oswestry Low Back Pain Disability Index) and NPDs (improving by 23.2% at Neck Disability Index) [23] and can improve motivation in physical activities [24].
**Neuro Rehabilitation**	VR and interactive video gaming were no more beneficial than conventional therapy approaches in improving upper limb function in stroke. VR may be beneficial in improving upper limb function and activities of daily living function when used as an adjunct to usual care [12].	The metaverse can be more effective than conventional therapy in patients with cerebral palsy in improving gross motor functions [22].
**Cognitive Rehabilitation**	It has been effectively used in psychiatric treatments, especially for anxiety disorders like specific phobias and post-traumatic stress disorder. This is because VR can enhance exposure therapy, a treatment based on fear-conditioning models aiming to reduce conditioned fear responses [13].	The metaverse and algorithm-based platform, designed to prevent cognitive decline, offers early interventions by training the brain, stimulating neural functions, and repairing memory. Enhanced communication between specific brain regions improves reasoning, response times, and overall cognition. The platform, incorporating gaming elements, has been effective in treating various neuropsychological issues, from cognitive decline and PTSD to Parkinson’s disease [36].
**Psychological Rehabilitation**	Virtual environments can be used to treat phobias, PTSD, anxiety disorders, psychosis, addiction, eating disorders, and disorders associated with child and adolescent psychiatry. By gradually exposing patients to the source of their fears or traumas in a controlled setting, therapists can guide them in developing coping mechanisms [14].	The metaverse can stimulate memory functions and motor activities (i.e., cooking) in patients with intellectual disability. However, there were no significant differences in the FAB total score (conceptualization, mental flexibility, motor programming, sensitivity to interference, inhibitory control, and environmental autonomy) [21].
**Social Integration**	The Multiplayer Rehabilitation Gaming System influences psychosocial dynamics by changing the participants’ mutual perception [15].	The metaverse provides a platform for social interaction. This can be particularly valuable for individuals with mobility issues or social anxieties, as it allows them to practice social skills and build connections in a less intimidating environment [18].
**Real-time Feedback**	Many VR rehabilitation tools offer real-time patient performance feedback, enabling therapists to adjust the difficulty and tailor the experience to individual needs.	On average, 97.9% of elementary school students had experiences with the metaverse, with 95.5% considering it closely related to their everyday life [17].

Virtual reality, VR; Non-specific low back pain, NS-LBP; Neck pain disorders, NPDs; Post-traumatic stress disorder, PTSD; Frontal Assessment Battery, FAB.

While advanced methods like VR may initially incur higher costs than traditional physiotherapy, their efficacy can offset these expenses. Integrating VR into standard rehabilitation practices was shown to be cost-effective when scaled across multiple patients, offering additional benefits and enhancing patient motivation [32,37]. Indeed, the metaverse can deliver rehabilitation services to remote or underserved areas, overcoming geographical barriers. Virtual environments can host multiple patients simultaneously, enabling group therapy. For instance, the use of a Back Extension-Virtual Reality Game (BE-VRG) proved to be more cost-effective for chronic non-specific low back pain compared to clinic-based McKenzie therapy (CBMT) [38]. Despite the economic implications, as described by Wang et al. [6], the metaverse offers unparalleled interactive experiences with peers, avatars, and AI agents. potentially revolutionizing the rehabilitation landscape.

In general, it can be said that the generation of a metaverse ecosystem [6], applied to the rehabilitation field, can present enormous power of diffusion on a global scale (scalability). In addition, the metaverse could not only act effectively on the rehabilitation of patients suffering from various diseases, but also reduce the economic costs of health services, being able to treat several patients at the same time and even remotely with telerehabilitation programs.

### 4.3. Metaverse and AI: Future Perspectives

The metaverse, integrated with artificial intelligence, offers transformative potential in medical and rehabilitation fields. Wang et al. [6] envision the potential for diagnostic interventions, prognosis assessments, and treatments directly within the metaverse, facilitated by continuous real-time data exchange through the internet—called the metaverse of “Medical Technology and Artificial Intelligence” (MeTAI). With AI-driven radiological image analysis, machine learning, and high-speed internet (5G/6G), this integration could revolutionize medical practices and enhance efficiency. Concurrently, several studies have already demonstrated the opportunities for using AI in the medical fields [39], such as diabetes [40,41] and musculoskeletal disorders [42,43], emphasizing the increasing potential for growth and innovation. The metaverse offers physicians immersive training sessions to simulate muscle pathologies, enhanced with integrated radiologic tools.

The metaverse holds potential in orthopedic surgery, especially in robot-assisted total arthroplasty, enhancing surgical precision. Postoperatively, it can revolutionize rehabilitation, allowing patients to undergo guided therapy in immersive environments, possibly supervised by AI or virtual therapists. The metaverse’s ability to simulate real-world distractions through dual-task training can bolster multitasking abilities vital for everyday life and sports. This integration challenges traditional rehabilitation approaches, promising more dynamic and efficient methods also in terms of telerehabilitation [26,44].

Indeed, as a virtual environment, the metaverse is well suited to serve as the primary platform for telerehabilitation sessions. In this context, its role would be to enable remote interventions for one or multiple patients simultaneously, thereby reducing healthcare costs. Telerehabilitation has proven to be particularly useful during the COVID-19 pandemic, with examples of remote virtual treatments for stroke patients; these interventions were shown to be effective and cost-efficient, making them easily adaptable to the metaverse platform [45].

Furthermore, the metaverse offers an innovative approach with a transformative role in several ways: 3D visualization of molecular interactions, manipulation, and analysis of molecular structures in real time, making the data interpretation process more efficient. In this scenario, various software applications leverage VR to assist physicians and ensure data accuracy.

As the metaverse can be expensive and inaccessible to some, no one has yet conducted a cost–benefit assessment. Furthermore, the limited number of studies with heterogeneous results hinders a precise quantitative evaluation and comparison of outcomes. There are also potential pitfalls to consider. One concern is the accessibility of the technology, as not everyone may have access to the necessary equipment or knowledge to use it. Additionally, some patients may have a learning curve in navigating the virtual environment, which could impede progress. Another consideration is the potential for addiction or over-reliance on the virtual environment, which could negatively impact real-world functioning. Additionally, there may be ethical concerns regarding using virtual reality for rehabilitation, such as the potential for privacy violations or the need for informed consent. The metaverse’s potential is vast, yet much remains to be explored. One critical aspect is translating virtual environment results into real-world improvements, with future studies focusing on this transition could provide valuable insights. Lastly, we know that the term “metaverse” is not always used in scientific papers using VR or AI.

## 5. Conclusions

The use of the metaverse in rehabilitation has shown promising results, with studies indicating improvements in physical and cognitive function and increased motivation and engagement. For example, stroke patients who participated in virtual reality-based rehabilitation showed more remarkable improvement in their upper limb function compared to those who received conventional therapy.

Then, the metaverse, a cutting-edge digital realm, represents a fascinating latest-generation reality and holds promising potential for its application in rehabilitation through the varied uses of VR/AR. It could undoubtedly be helpful in the rehabilitation treatment of various pathologies. Despite the fact that the term “metaverse” was only defined in 2021, the studies included in this narrative review showed its promising efficacy in several rehabilitation domains, such as therapeutic exercise, cerebral palsy intervention, and treatment of intellectual disabilities. However, it is premature to conclusively determine the superiority of the metaverse over traditional rehabilitation methods due to the limited sample size of current patients across studies.

There is a noticeable scarcity of high-impact studies (like RCTs) exploring rehabilitation realized through the metaverse. Furthermore, future research should aim to contrast the efficacy of metaverse-based rehabilitation with interventions using conventional VR/AR and placebo-controlled VR/AR.

Overall, while using the metaverse in rehabilitation shows promise, it is essential to carefully consider the potential benefits and drawbacks before implementing it as standard practice.

## Figures and Tables

**Table 1:** Characteristics of included studies on metaverse applications in rehabilitation.

Reference	Study Design	Target Conditions	Metaverse Platform	Aim of the Study	Duration of the Intervention	Patient Count	Key Findings	Limitations of the Studies
Cheung et al. (2022)	RCT	Intellectual disability	VR wearable technology with human–virtual world interaction	Comparison of efficacy between three arms: (1) rehabilitation in the virtual world, (2) traditional rehabilitation, and (3) control (no intervention)	One day	145: VR (n = 42); traditional (n = 53); and control (n = 50)	VR had significantly larger improvement effects in cooking (cooking score with Wald *χ*^2^ = 4.253; *p* = 0.039) than the control group and memory span compared to traditional training (Wald *χ*^2^ = 4.235; *p* = 0.040) and control group (Wald *χ*^2^ = 4.210; *p* = 0.040).	- Risk of bias (per protocol analysis approach); - Lack of validation for the rating scales; - No complete diagnosis information (for Down’s syndrome and autism spectrum disorder); - IQ is a confounding factor.
Mizuta et al. (2024)	RCT	Healthy young people	Metaverse space	Comparison of efficacy between three arms: the metaverse, YouTube, and control group without instructions	8 weeks of exercises	48: VR (n = 16); YouTube (n = 16); control group (n = 16)	Significant interaction between groups and before and after the intervention for total physical activity (*p* = 0.04) but none for the indicators of well-being (*p* = 0.40), locomotive function scale (*p* = 0.17), and social capital (*p* = 0.23). A post hoc test showed a significant increase in physical activity in the metaverse group before and after the intervention (*p* = 0.006).	- The target population were students interested in exercising; - It was difficult to accurately determine the time and frequency of interactions between participants in the metaverse space.
Moon et al. (2023)	RCT	Cerebral palsy	Metaverse physical therapy	Comparison of efficacy between MPT and CPT	Three days/week for four weeks	26: MPT (n = 13); CPT (n = 13)	The results provide promising therapeutic evidence that MPT improves GMFM-66 (*p* = 0.001), cardiopulmonary function with HR (*p* = 0.03) and BRPE (*p* = 0.001), and the risk of COVID-19 in children with CP (*p* = 0.001)	- Lack of follow-up evaluation; - No comparison between GMFCS and gait ability; - Possible differences in movement recognition caused by the quality of the smartphone or tablet camera.
Orr et al. (2023)	Retrospective study	Non-NS-LBP and NPDs	Exercise therapy delivered solely in the metaverse using virtual reality	To show the efficacy of the use of metaverse in patients with NS-LBP and/or NPDs	The mean of 127.1 days of treatment	82 with NS-LBP and/or NPDs	Disability from NS-LBP was significantly reduced when assessed with the Modified Oswestry Low Back Pain Disability Index by 17.8% (*p* < 0.001) and from NPDs (Neck Disability Index) by 23.2% (*p* = 0.02)	- No randomization; - No blinding; - No comparison with a non-treatment group; - Lack of equal characteristics of the population in the three countries.
Shah et al. (2022)	Prospective study	Elderly with a reduction in physical strength due to physical inactivity	Social VR-based collaborative exergame	To show the efficacy of metaverse stimulating physical exercise and improving social connectedness during rehabilitation	Five weeks	14: playing collaboratively (n = 7); playing individually (n = 7)	The participants exergaming collaboratively in the fourth week reported significantly higher intrinsic motivation on all subscales (enjoyment: *p* < 0.02; effort: *p* < 0.002; usefulness: *p* < 0.01) and physical exertion (*p* < 0.001) than those playing alone	- Limited number of participants (n = 14); - Short span of play time (five weeks); - Lack of control over patients’ health status; - No randomization.

Metaverse physical therapy, MPT; Conventional physical therapy, CPT; Gross Motor Function Measure, GMFM-66; Heart rate, HR; Borg Rating of Perceived Exertion, BRPE; Gross Motor Function Classification System, GMFCS; Cerebral palsy, CP; Non-specific low back pain, NS-LBP; Neck pain disorders, NPDs.

**Table 2:** Virtual reality versus metaverse.

Side-by-Side Comparison	Virtual Reality	Metaverse
**Definition**	VR is a digital environment where users can interact with special VR headsets. This environment can be entirely computer-generated or a real-world environment captured and rendered digitally. VR is an immersive experience where all visual stimuli come from the digital environment.	Metaverse describes a collective virtual shared space created by converging virtually enhanced physical reality with interactive digital spaces. It includes AR, VR, and the internet. Think of the metaverse as an expansive digital universe comprising many interconnected virtual environments and public spaces.
**Interactivity**	It offers a fully immersive experience. Users might interact with the environment using hand controllers, motion sensors, and voice commands.	It provides a higher degree of interaction, not just with the environment but also with other users in real-time. It is more socially connected, often with avatars representing users and facilitating interactions.
**Scope**	Typically, it is limited to the specific environment or application with which the user is engaged. It is a component of the metaverse but does not encompass its entirety.	It is a broader, interconnected universe of digital spaces. It includes VR environments but extends beyond them to incorporate AR, 3D internet spaces, and more.
**Purpose**	It is often designed for specific applications such as gaming, training, education, or therapy.	It is envisioned as an expansive digital universe for myriad activities (socializing, commerce, gaming, work, and more).
**Accessibility**	It requires specific hardware (like VR headsets) and can be more restrictive regarding access and cost.	It aims to be universally accessible from various devices and platforms, including VR headsets, computers, smartphones, and AR glasses.

Augmented reality, AR; Virtual reality VR.

## Data Availability

Not applicable.
